# Classifying the evolutionary and ecological features of neoplasms

**DOI:** 10.1038/nrc.2017.69

**Published:** 2017-09-15

**Authors:** Carlo C. Maley, Athena Aktipis, Trevor A. Graham, Andrea Sottoriva, Amy M. Boddy, Michalina Janiszewska, Ariosto S. Silva, Marco Gerlinger, Yinyin Yuan, Kenneth J. Pienta, Karen S. Anderson, Robert Gatenby, Charles Swanton, David Posada, Chung-I Wu, Joshua D. Schiffman, E. Shelley Hwang, Kornelia Polyak, Alexander R. A. Anderson, Joel S. Brown, Mel Greaves, Darryl Shibata

**Affiliations:** 1 Virginia G. Piper Center for Personalized Diagnostics, Biodesign Institute, Arizona State University, 1001 S. McAllister Ave, Tempe, Arizona 85287, USA; 2 Department of Psychology, Center for Evolution and Medicine, Arizona State University, 651 E. University Drive, Tempe, Arizona 85287, USA; 3 Evolution and Cancer Laboratory, Centre for Tumour Biology, Barts Cancer Institute, Queen Mary University of London, Charterhouse Square, London EC1M 6BQ, UK; 4 Centre for Evolution and Cancer, The Institute of Cancer Research, 15 Cotswold Road, Sutton, London SM2 5NG, UK; 5 Department of Anthropology, University of California Santa Barbara, Santa Barbara, California 93106, USA; 6 Department of Medical Oncology, Dana–Farber Cancer Institute, 450 Brookline Avenue D740C, Boston, Massachusetts 02215, USA; 7 Department of Cancer Imaging and Metabolism, Moffitt Cancer Center and Research Institute, 12902 Magnolia Drive, Tampa, Florida 33612, USA; 8 Centre for Evolution and Cancer, Division of Molecular Pathology, The Institute of Cancer Research, 237 Fulham Road, London SW3 6JB, UK; 9 Brady Urological Institute, The Johns Hopkins School of Medicine, 600 N. Wolfe Street, Baltimore, Maryland 21287, USA; 10 Cancer Biology and Evolution Program, Moffitt Cancer Center, 12902 Magnolia Drive, Tampa, Florida 33612, USA; 11 Cancer Research UK Lung Cancer Centre of Excellence, University College London Cancer Institute, Paul O’Gorman Building, 72 Huntley Street, London WC1E 6BT, UK; 12 Department of Biochemistry, Genetics and Immunology and Biomedical Research Center (CINBIO), University of Vigo, Spain; Galicia Sur Health Research Institute, Vigo, 36310, Spain; 13 Department of Ecology and Evolution, University of Chicago, Chicago, Illinois 60637, USA; 14 Departments of Pediatrics and Oncological Sciences, Huntsman Cancer Institute, University of Utah, 2000 Circle of Hope, Salt Lake City, Utah 84108, USA; 15 Department of Surgery, Duke University and Duke Cancer Institute, 465 Seeley Mudd Building, Durham, North Carolina 27710, USA; 16 Integrated Mathematical Oncology Department, Moffitt Cancer Center, 12902 Magnolia Drive, Tampa, Florida 33612, USA; 17 Department of Pathology, Norris Comprehensive Cancer Center, University of Southern California, 1441 Eastlake Avenue, NOR2424, Los Angeles, California 90033, USA

## Abstract

Neoplasms change over time through a process of cell-level evolution, driven by genetic and epigenetic alterations. However, the ecology of the microenvironment of a neoplastic cell determines which changes provide adaptive benefits. There is widespread recognition of the importance of these evolutionary and ecological processes in cancer, but to date, no system has been proposed for drawing clinically relevant distinctions between how different tumours are evolving. On the basis of a consensus conference of experts in the fields of cancer evolution and cancer ecology, we propose a framework for classifying tumours that is based on four relevant components. These are the diversity of neoplastic cells (intratumoural heterogeneity) and changes over time in that diversity, which make up an evolutionary index (Evo-index), as well as the hazards to neoplastic cell survival and the resources available to neoplastic cells, which make up an ecological index (Eco-index). We review evidence demonstrating the importance of each of these factors and describe multiple methods that can be used to measure them. Development of this classification system holds promise for enabling clinicians to personalize optimal interventions based on the evolvability of the patient’s tumour. The Evo- and Eco-indices provide a common lexicon for communicating about how neoplasms change in response to interventions, with potential implications for clinical trials, personalized medicine and basic cancer research.

Neoplasms evolve^1–3^. This evolution has been recognized since 1976 (REF. 4), and it explains the processes of both carcinogenesis and acquired therapeutic resistance^1^. The evolution of neoplasms is shaped by the selective pressures of their microenvironmental ecology. But between and within cancer types, tumours probably display differences in the dynamics of cancer evolution and ecology, including the rates at which new clones appear and go extinct, how different those clones are from one another and whether they appear in bursts or at a more regular pace. Many of the evolutionary and ecological properties of a neoplasm are clinically relevant^5–16^, though this is not always true^6,16,17^, and in most cases their clinical relevance has not yet been tested. There is a need for a common language and conceptual categories for drawing clinical distinctions that capture the relevant genetic, environmental and kinetic parameters that impact tumour adaptation and progression, as well as response to therapy. A classification system for the evolution and ecology of neoplasms would provide clinicians and researchers with a foundation for developing better prognostic and predictive assessments of tumour behaviour, such as response to an intervention.

The ultimate purpose of a classification system for the evolution and ecology of neoplasms is to provide a descriptive tool by which to improve clinical management with respect to the overall survival and quality of life of the patient. It would also help to drive research and discovery in cancer biology and oncology.

Below, we discuss the methods by which we reached consensus as well as the goals and guiding principles we aspired to in the development of a framework for classifying neoplasms. We then discuss each of the components of the classification system as well as methods for measuring them and for dividing tumours into an initial set of 16 classes. We discuss how such a classification system could be developed, improved and used clinically in the future.

## Methods

We convened a consensus conference of experts in the fields of cancer evolution and cancer ecology to lay the groundwork for the development of an evolutionary and ecological classification system. The initial participants (Maley, Aktipis, Graham, Sottoriva, Boddy, Janiszewska, Silva, Gerlinger, Anderson, Brown and Shibata) were among the faculty for the Evolution and Ecology of Cancer summer school funded by Wellcome and held at the Wellcome Genome Campus in Hinxton, UK, in July of 2016. Input from all participants was solicited, and after discussion, we identified areas of consensus. Afterwards, other leaders in the field were invited to join the effort by co-editing and discussing the developing statement. All authors reviewed and approved the final statement. Wellcome Genome Campus Advanced Courses and Scientific Conferences provided financial support for the consensus meeting. We have named the classification system, with their permission, in appreciation of Wellcome’s support. Please note that the statement reflects the opinions of the authors and not necessarily those of Wellcome.

## Goals and guiding principles

Our development of this framework has been guided by several goals and principles. We agreed that an ideal classification system should have the following properties. First, it must be able to alter a clinical decision point. Second, it should be simple enough to be easily remembered and applied. Third, it should also align with our current understanding of the dynamics of neoplasms. Fourth, the classification system should be general enough to be applied across different types of neoplasm, recognizing that the types of measurement may need to be individualized to a given type of cancer.

This framework is based on fundamental theoretical principles underlying evolutionary and ecological dynamics. It is not based on any particular assay or parameter but rather captures the fundamental drivers of tumour evolution. This is a necessary first step that we hope will lead to many methodological and measurement innovations to quantify the key components of tumour evolution and ecology that we identify here. Because the evolution of cancer is still a relatively new field, there is still uncertainty about the best ways to measure and describe the evolution and ecology of a tumour.

There are also practical considerations in the construction of a classification system. If a tumour could be classified based on a single biopsy from standard assays such as those that can be done on formalin-fixed paraffin-embedded (FFPE) tissue or standard radiological images, translation to the clinic would be relatively easy. However, studies have not yet been done to test whether measures of the evolvability of a tumour from a single biopsy sample are sufficient or whether multiple samples substantially improve predictions of clinical outcomes^15^. We hypothesize that we will need to extensively sample neoplasms over both space and time in order to accurately quantify their evolvability, but this remains an open question. It is clear, however, that evolutionary analyses are limited if the clonal structure of the primary tumour is unknown^18^. The use of cell-free DNA (cfDNA) from liquid biopsy samples should facilitate longitudinal studies^19^, although deconvoluting the clones within such a mixed sample remains a challenge^20^.

## Framework for classifying tumour evolution

There are many well-established ways to classify tumours, largely based on extent of spread and morphological appearances (for example, stage and grade). An evolutionary classification system would augment current schemes by further capturing the evolvability of a tumour. How much intrinsic genetic instability does it have? How likely is it to respond quickly to a new selective pressure such as a therapeutic intervention? For example, rapid progression after chemotherapy is probably driven by pre-existing resistant variants, and therefore, failure is more likely in tumours with more subclonal diversity (intratumoural heterogeneity)^6^. Moreover, it would be useful to classify evolution through time. For example, a second biopsy from the same patient after therapy may reveal minimal diversity, indicating a recurrent tumour derived from a single clone, or substantial diversity, suggesting intrinsic resistance by the majority of tumour cells. There was widespread agreement at the consensus conference that both the evolutionary dynamics of the neoplastic cells themselves (cancer cell intrinsic factors) and the microenvironment that defines the ecology of those cells (cancer cell extrinsic factors) are important in predicting the future behaviour and response of a tumour. To capture this, we have developed a framework for both an evolutionary index (Evo-index) that describes the intrinsic evolvability of the neoplastic cell population and an ecological index (Eco-index) that describes potential selective pressures imposed by the surrounding microenvironment.

## The Evo-index

**Figure 1:**
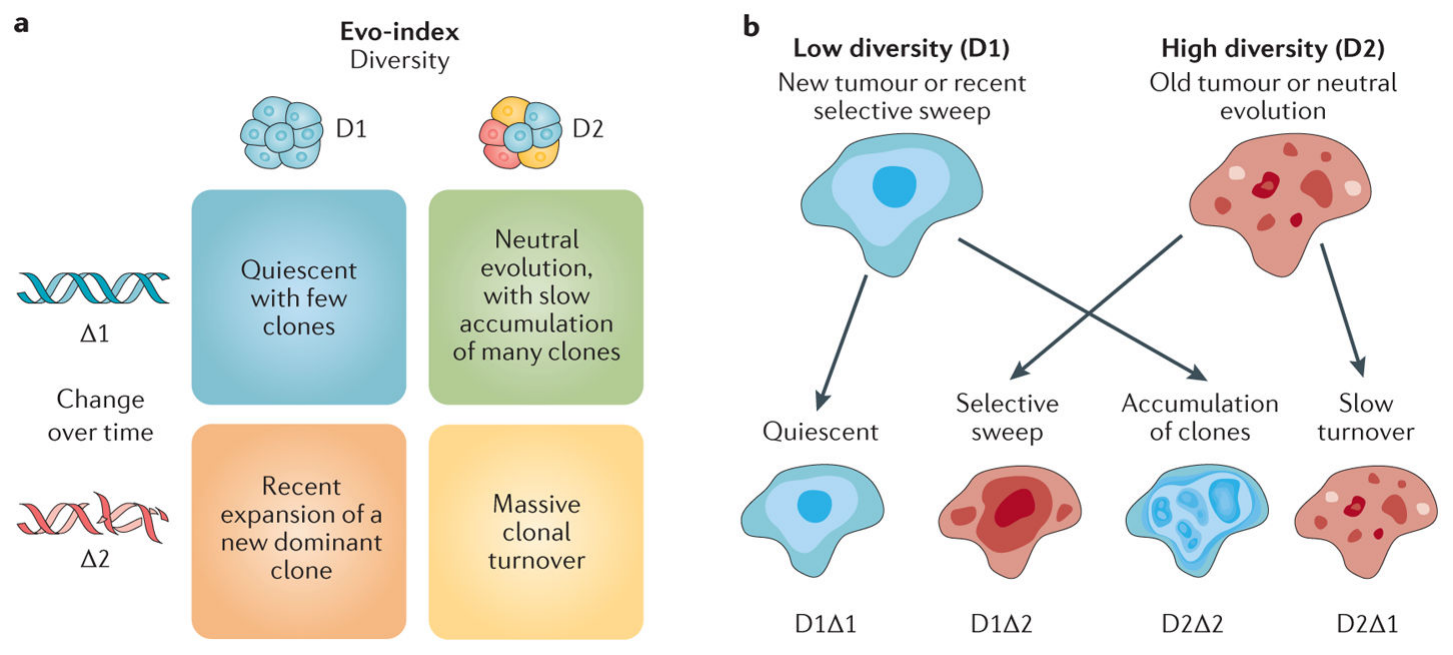
The Evo-index and how it changes **a** | The evolutionary index (Evo-index) is composed of two factors corresponding to heterogeneity over space (diversity, D) and heterogeneity over time (change over time, Δ). By ‘change’, we mean both change in the genetic, epigenetic and phenotypic alterations present in the population and change in the frequencies of those alterations in the neoplastic cell population. What measures of D and Δ are best is an open question. In addition, how these factors should be stratified into two, three or more classes is also an open question. Here, for simplicity, we provide examples of the kinds of dynamics that could be categorized into a simple 2 × 2 classification. **b** | The genetic composition of a tumour may change either slowly (Δ1) or rapidly (Δ2) in a variety of ways. On the left, a tumour may have low diversity (D1) at time 0 because it is a new tumour or there has been a recent homogenizing clonal expansion. That tumour may be quiescent and so appear substantially the same at time 1 (D1Δ1), or it may accumulate clones, some of which expand, to generate a diverse tumour by time 1 (D2Δ2). Alternatively, a tumour may be diverse (D2) at time 0 because it is old or has a high mutation rate and is evolving neutrally. At time 1, that tumour may have been homogenized by a selective sweep (D1Δ2) or may continue on its current trajectory with gradual turnover of its clones (D2Δ1).

The Evo-index (D#Δ#) is a combination of two fundamental components: the diversity (D) or intratumoural heterogeneity of the neoplasm and how it changes over time (Δ). In other words, the Evo-index quantifies heterogeneity in both space and time (FIG. 1a). Both diversity and changes in the clonal structure of a tumour over time are objective measures and may be assessed as part of preclinical studies or clinical trials.

### Diversity

The heterogeneity that is currently present in a population defines its capacity to respond, at a population level, to selective pressures. This diversity is the fuel for the engine of natural selection. There are different forms of diversity, including genetic diversity, epigenetic diversity, phenotypic diversity and functional diversity. Genetic diversity can predict progression to invasive cancers^12,13^ as well as recurrence and survival^5–9,16^. The relationship between diversity and clinical outcomes is not universally consistent across different cancer types^6,16^ and can be complicated (BOX 1).

Box 1 **Important issues in the measurement of diversity in neoplasms**

There are a number of important issues and open questions in the measurement of diversity in neoplasms: How are clones defined? What is the best measure of diversity? How do the measures scale up to genomic assays? Are there nonlinear associations between diversity and clinical outcomes? Is genetic or functional diversity more predictive? Is it sufficient to measure diversity in the primary tumour, or do we need to measure diversity in the metastases? Is it adequate to estimate diversity from bulk biopsy assays, or do we need to measure diversity at the single-cell level?

In order to measure diversity, one must first define the unit that is being measured. We typically cluster cells into clones, but there is currently no general definition of a clone. Typically, for expediency, clones are defined as the set of cells that share an alteration of interest, due to descent from a common ancestor cell. A more stringent definition of a clone is a set of cells that have the same genotype based on some assay^12,13^. However, that definition does not scale well to whole-genome assays because every neoplastic cell probably displays a unique genome. By contrast, measures of divergence between samples only become more accurate as assays scale up to the genomic level^12–14,46^. Another alternative would be to reconstruct the cell lineage (phylogeny) of a neoplasm and then define clones based on the topology of the cell lineage, although this is not straightforward. A similar problem has been addressed by viral and bacterial phylogenetics, and methods may be borrowed from these fields^167,168^.

It is not clear which alterations should be used to measure diversity. Some forms of diversity, such as mutations in exons and copy number changes, may be more clinically relevant than other forms of diversity. However, Merlo and colleagues found that defining a clone based on selectively advantageous mutations and defining a clone based on evolutionarily neutral mutations both predicted progression to cancer^13^.

Instead of genetics, one could measure diversity based on RNA expression or other phenotypic characteristics^169,170^. Because selection acts on phenotypes, this may be a better predictor of a the evolvability of a tumour than genetic measures of diversity. Gatenby and colleagues have argued that because of this and the fact that there are many different genotypes that can produce the same phenotype, analysis at the phenotype level may be easier and provide a better measure of evolvability than analysis at the genotype level^171,172^. However, this hypothesis is controversial, and only a few studies have tested it^173,174^. Unfortunately, the literature on how to measure functional diversity remains poorly developed^30^.

The diversity of the primary tumour may differ from that of any metastases. Because the primary tumour is often removed and it is the metastases that kill patients, we may have to measure diversity within and between any metastases that can be sampled in order to best predict clinical outcomes^2^.

It is currently difficult to measure many loci or phenotypes at the single-cell level. Bulk sequencing or other assays at the biopsy level introduce significant biases. For example, recent mutations that are present in only a single cell or a small minority of cells are missed in bulk assays, biasing results to the early mutations and those mutations driving clonal expansion^175^. Preliminary analyses show that a mixture of clones within a biopsy sample can also mislead any analyses based on estimates of shared ancestry, such as phylogenetic reconstruction^176^. However, it is currently difficult to assay enough loci in enough single cells to reconstruct reliable cell lineages and identify rare clones^177^.

Diversity can be a proxy for the likelihood that a resistant clone is present in a neoplasm. We currently do not know all the mutations and epigenetic alterations that make a neoplastic cell resistant to a particular therapy, and even those we do know are difficult to detect if they are present in only a small region of the tumour. Compared with homogeneous neoplasms, diverse neoplasms are more likely to harbour resistant clones and are also probably more likely to evolve resistance in the future.

Multiple forms of diversity within a neoplasm may be clinically important, not only as fuel for natural selection but also as biomarkers of clinically targetable dynamics. For example, if high levels of genetic diversity are indicative of high levels of moderately deleterious passenger mutations^21,22^, then suppressing mechanisms in the cell that buffer against those deleterious effects, such as chaperone proteins, should preferentially harm neoplastic cells^21^. Alternatively, diversity may be indicative of cooperation between clones, through mechanisms such as cross feeding^23–27^. These mechanisms of cooperation are themselves potential therapeutic targets. Theory suggests that targeting cancer cell cooperation should provide weaker selection for resistance than cytotoxic therapies^28^.

It is likely that not all forms of diversity are equal, and future work must test which are clinically relevant. It may be the case that measures of functional diversity or even phenotypic diversity are better predictors of clinical outcomes than measures of genetic diversity (as many genetic mutations will have no phenotypic consequence), and the ideal measures may vary between tumour types.

### Measuring diversity

Of the four components of the classification framework, the largest number of methods has been developed for measuring diversity (intratumoural heterogeneity)^13,25,29^ (TABLE 1). There is a large literature in ecology on the quantification of diversity^30^. The overall diversity of a large area, or landscape (gamma diversity), can be broken down into the diversity within local regions (alpha diversity) and the differences between regions (beta diversity)^31^. Inherent in this definition is the concept that measuring diversity requires defining the spatial scale that one is examining. One might define within-region diversity as the diversity measured within a biopsy sample**,** while between-region diversity would account for differences between biopsy samples in multi- region sampling studies. Alternatively, one could take a sample across an entire tumour, perhaps using cfDNA, and estimate the diversity of the entire population. Most of the studies to date have focused on within-region diversity^5,6,32,33^ or the diversity of the entire tumour^12–14,25^. The use of ecological statistics for measuring between-region diversity in tumours remains relatively unexplored. Established measures of differences between microbial communities^34^ could possibly be applied to measuring differences between biopsy samples.

There are many ways to measure diversity^30^ and a number of challenges to measuring diversity in neoplasms, as discussed in BOX 1. In Barrett oesophagus, Merlo and colleagues tested many of those measures of diversity and found that high levels of diversity were predictive of progression to cancer, regardless of the measure^13,14^. Because evolution is driven by the fitness outliers^35^, and it may take only one resistant cell at diagnosis to eventually cause drug resistance or relapse after therapy, much of the predictive value of measuring diversity may lie in the long tail of rare clones. Because of this, we recommend using either a count of the number of clones (‘species richness’) or Shannon index, which equally weights number and relative abundance of clones, to quantify diversity^30^.

The feasibility of obtaining a complete picture of the diversity of a neoplasm, through multi-region sampling or cfDNA, varies across tumour types. In Barrett oesophagus, bladder cancer and prostate cancer, multi-region sampling is part of the current standard of care^36–38^. In a well-mixed neoplasm, such as a blood cancer, a single sample may be sufficient, but it requires single-cell assays, which have their own challenges (BOX 1). In other tumours that are difficult to sample, such as pancreatic cancers, we are lucky to get more than one biopsy sample. The main challenge in using cfDNA is detecting it in serum for cancers that have not yet metastasized, although the level of tumour cfDNA in serum varies across cancer types. A recent study was able to detect tumour cfDNA in 97% of early-stage lung squamous cell carcinomas but only 19% of early-stage lung adenocarcinomas^39^.

The interpretation of the diversity of a neoplasm depends on the context of its history. A neoplasm that has just been homogenized by a therapy that killed most of the clones in that neoplasm is different from a neoplasm that is homogeneous because it has a very low mutation rate and has not had enough time to accumulate many clones. By contrast, a high level of diversity in a neoplasm that has just passed through a therapeutic bottleneck may be a sign that therapy selected for a mutator phenotype^40^. Because of this complication, we agreed that we must measure how neoplasms are changing over time as well as diversity.

### Change over time

There are a variety of ways that a neoplastic cell population changes over time. These include mutations, natural selection and genetic drift. One important parameter of change over time is the mutation rate, which describes how fast a lineage accumulates new mutations. Of course, there are different mutation rates induced by each mechanism for genetic and epigenetic alteration, including mutation signatures induced by specific agents^41^ as well as telomere erosion, non-homologous recombination, other forms of chromosomal instability, CpG methylation and his-tone modifications. Which mechanisms are relevant will depend on individual tumours and may vary across the different clones within the same tumour.

When we talk about and measure mutation rates, we are implicitly assuming that mutations happen at a regular rate. Evolutionary biologists call these ‘molecular clocks’ (REF. 42). However, a catastrophic mitosis can generate chromosomal alterations across the genome in a single event^43,44^. There is a continuum from regular, gradual, clock-like small alterations to sporadic, punctuated, large alterations. For example, a lineage may evolve different mutation rates across its history, as happens with the evolution of a mutator phenotype^45,46^. If a cell lineage can change suddenly, in what used to be called a ‘macromutation’ generating a ‘hopeful monster’ (REF. 47), then that tumour may have a different capacity for evolution compared with a tumour that is constrained to evolve through the slow accumulation of mutations with small phenotypic effects. There is a large cancer literature on genetic instability that is relevant here^48,49^, and evidence has shown that tumours with extremely high mutation rates may have a better prognosis than tumours with moderate rates^6,11,21,22,50^. High levels of genomic instability may make it difficult for cell lineages to maintain the adaptive information encoded in their genomes, generating non-viable daughter cells, and may also produce an abundance of neo-antigens that stimulate an antitumour immune response^6^. Furthermore, high mutation rates of single nucleotide variants can generate deleterious mutations, leading to the fitness decline of neoplastic cell lineages in a form of Muller’s ratchet^21,51^. This may even cause tumour regression in some cases^21,22^.

The genetic composition of a population changes over time not only through the rate at which mutations arise and the genetic drift of those alleles but also through the action of natural selection. Natural selection leads to adaptations, such as drug resistance^52^, that are clinically relevant. Detecting and measuring natural selection is likely to be an important component of our future clinical management of cancers.

The classification of a neoplasm’s change over time (Δ) will probably need to take into account both the speed at which a tumour acquires genetic or epigenetic alterations, or changes phenotypically, including how fast clones spread by natural selection, as well as the tempo of that change (from gradual to punctuated). The appropriate intervals for longitudinal sampling will depend on the rate of change over time^53^. Note that neutral, or ‘passenger’, mutations should not be ignored in these calculations because selective pressures change over time, particularly with the onset of therapy. Thus, resistance mutations, which may be deleterious or neutral in the absence of therapy, can become selectively advantageous for neoplastic cells exposed to therapy^54^.

### Measuring change over time

Measuring change over time is complicated, whether it is genetic or phenotypic change (TABLE 1). FIGURE 1b illustrates a simple version of how the Evo-index can describe evolutionary changes in tumour cell populations. It is possible for there to be change over time but for diversity to remain stable, with a dynamic equilibrium of clones appearing and going extinct^14^. For single samples, past genetic changes over time can be indirectly inferred based on mutation frequencies^17,55^. Sottoriva and Graham have pioneered methods to infer the mutation rate and to distinguish between tumours that are dominated by genetic drift versus those with evidence of natural selection after transformation. In the absence of selection, mutations that occur in the first cell division after transformation should appear in approximately one-half of all cancer cells, mutations that occur in the second round of cell division should appear in one-quarter of all cancer cells, and so on^17,56^.

**Figure 2:**
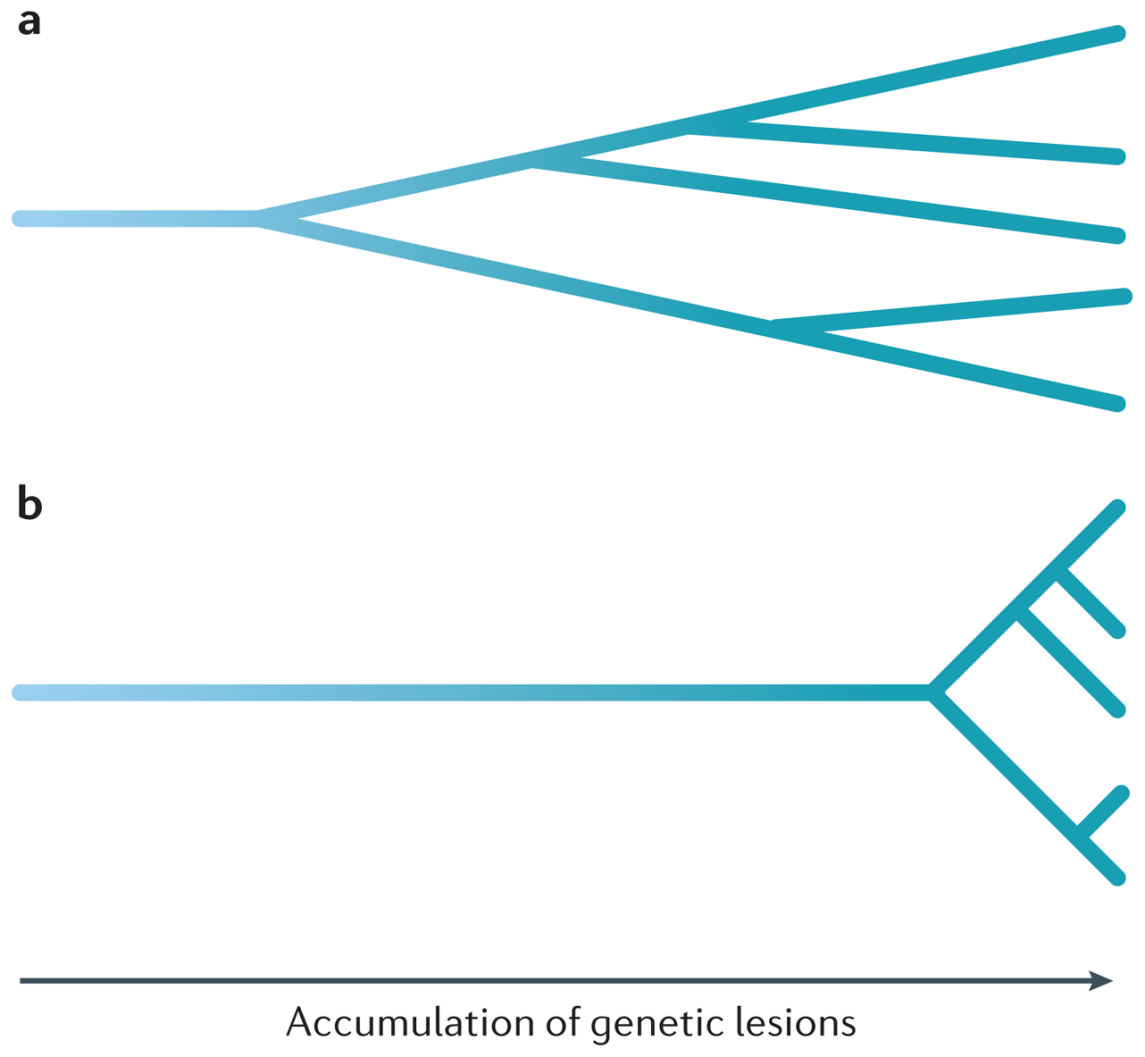
Clonal divergence is independent of clonal structure The cell lineages from two tumours may have the exact same clonal structure when they are sampled at the far right but have radically different degrees of genetic divergence. If one tumour (part **a**) has a higher mutation rate or has been accumulating genetic alterations for a longer period of time because those cells had a common ancestor, it will have a higher level of genetic divergence than another tumour (part **b**).

There are a number of measures of genetic change over time from population genetics that might be used on neoplasms, including Nei’s standard genetic distance^57,58^ and the Jaccard similarity coefficient^59^, as well as measures of beta diversity that can also quantify changes in a community over time, such as UniFrac^34^ or the fixation index^60^. The degree of genetic divergence between samples (called ‘nucleotide diversity’ in molecular population genetics) provides indirect information on the degree of change over time. Genetic divergence is often defined as the percentage of the genome that is different between pairs of samples^12–14^. This statistic provides predictive power independent of the number of clones for predicting progression^12,13^, supporting the framework of including both diversity and change over time in the Evo-index. Note that the same clonal structure can have radically different degrees of genetic divergence (FIG. 2). Maley and colleagues have calculated a mean pairwise divergence score between all pairs of samples from a neoplasm^12–14^. As the chance that two samples come from the same clone (and so have minimal divergence) depends on the size of the clone, the mean pairwise divergence blends the degree of divergence with clone size measures (and so blends D with Δ).

One of the primary tools for measuring change over time in evolutionary biology is phylogenetic inference, which reconstructs the history of a neoplasm^61,62^. Phylogenetic methods can be used to describe and quantify diversity patterns as well as rates of evolution across both space and time. Multiple phylogenetic approaches have been developed in recent years to study tumour evolution within a patient, both for bulk and single-cell data and from a variety of data types^20,63^. These methods depend on evolutionary models for the likelihood of molecular alterations occurring in neoplastic cell lineages, although the development of these models is still in its infancy.

All of the measures discussed so far can be calculated from a single timepoint. Of course, the degree and nature of change over time can be better measured directly with longitudinal samples. Minimally invasive assays, such as sequencing cfDNA from longitudinal blood samples, could reveal the action of natural or artificial selection in patients.

Incorporation of the Evo-index into clinical trials can better describe, in evolutionary terms, why interventions fail. Most human tumours at the time of clinical presentation contain multiple large clones^6,16^ and probably many more small clones^64,65^, and relapse without a reduction in diversity would probably imply intrinsic resistance or perhaps that an intervention resulted in increased mutagenesis. By contrast, relapse with less diversity (D1) implies a bottleneck effect where only a minority of tumour cells survived the intervention, probably indicating selection for one or a few resistant clones.

## The Eco-index

**Figure 3:**
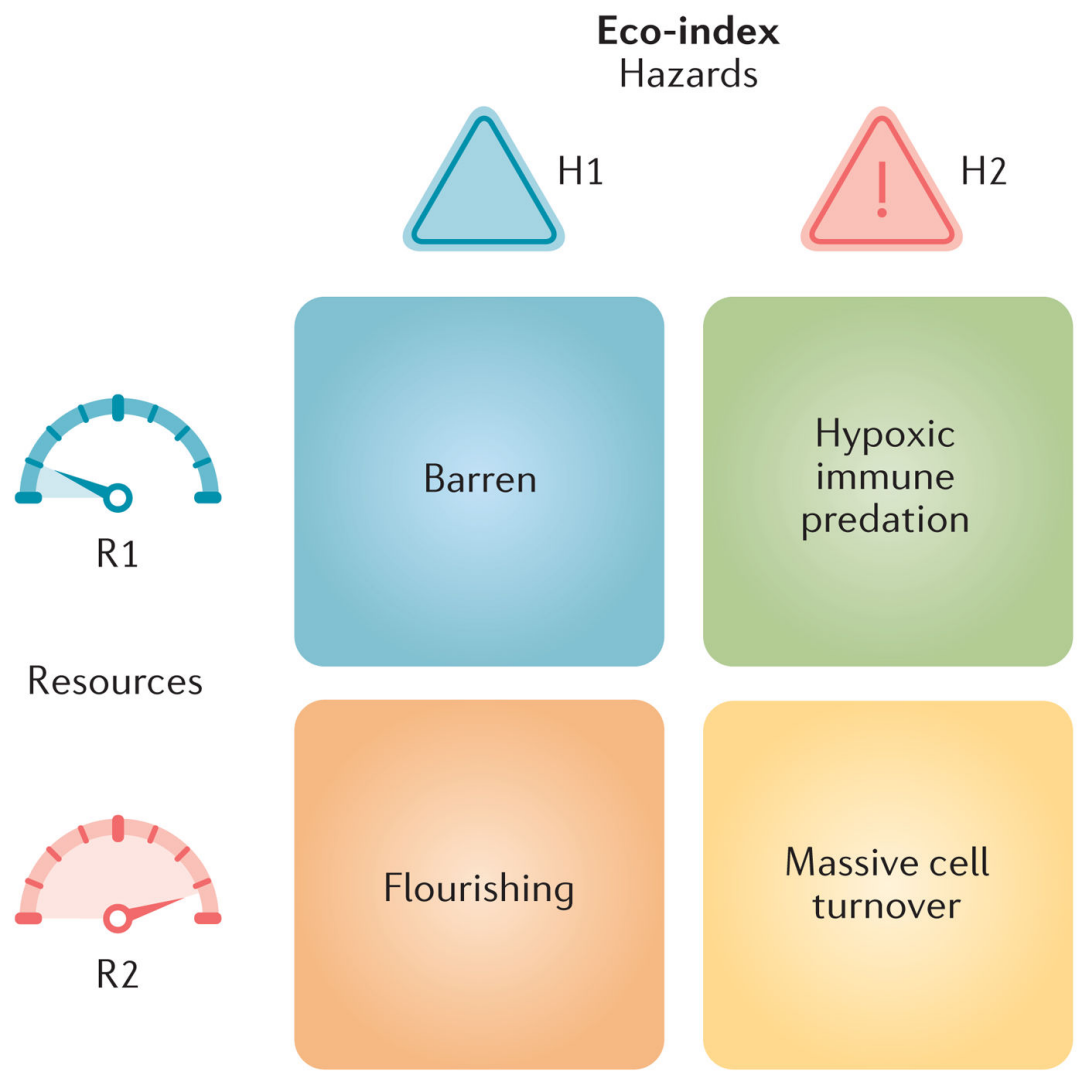
The Eco-index sThe ecological index (Eco-index) is composed of two factors corresponding to the hazards (H) and resources (R) available to the neoplastic cells. These capture the broad categories of selective pressures on a population. We have included example phenomena in this figure that might be observed in the different combinations of the degrees of hazards and resources. For example, a tumour with low hazards (H1) and low resources (R1) might be relatively barren, with few infiltrating lymphocytes but also poor perfusion and few supporting cells. Such an environment would select for cells that can either survive on few resources or move to locate more resources. High levels of hazards (H2) should, according to life history theory^71^, select for rapid proliferation, evasion of predation, migration away from the hazards^67^ and little investment in cell (and DNA) maintenance. High levels of resources allow neoplastic cells to rapidly proliferate. Thus, an H2R2 tumour would probably undergo massive cell turnover as cells are killed by the hazards and replaced by their rapidly proliferating sisters.

From the perspective of an organism or a neoplastic cell, its ecology can be broadly described by two characteristics: hazards (H) and resources (R)^66–69^ (FIG. 3). Hazards, here, are the things that can kill a cell. The relevant resources required for cell maintenance and growth are many and varied; whatever may potentially limit the growth of the neoplastic cell population^66^. Note that hazards and resources here are understood from the perspective of the neoplastic cell, not the patient. This is an important point from ecology —we can understand the evolution and responses of a population best when we take the perspective of an organism in that population^70^.

From an ecological perspective, the hazard and resource profiles for a species select for the particular life history strategies of that species. Aktipis and colleagues argued that the same principles are true for neoplastic cells^71^. Species that are exposed to high levels of hazard tend to evolve fast life history strategies, reproducing quickly and investing little in maintenance and survival. Organisms subjected to hazards generally leave behind higher levels of unexploited resources. Ecosystems with high or fluctuating resource supplies favour organisms that can rapidly reproduce to exploit those opportunities. This selects for speed over efficiency and can result in very high population densities but also fluctuating levels of unexploited resources. By contrast, populations that have few hazards and a steady supply of resources will tend to expand to the carrying capacity of the habitat, at which point natural selection favours organisms that can best compete for and efficiently utilize the limiting resources^72^. The heterogeneity of resources and hazards across space also has important impacts on the future evolution of cancer cell populations and prognosis for patients^73,74^.

### Hazards

There are multiple sources of hazards for neoplastic cells, including immune cells, toxins, waste products, microorganisms and anticancer therapies. There is good evidence that immune predation is associated with improved cancer prognosis^73,75–83^. Furthermore, there is emerging evidence linking high mutation loads that result in the formation of neo-antigens with immune predation and better survival in patients treated with immune checkpoint blockade therapies^84–86^. In addition, a high subclonal neo-antigenic burden is associated with worse outcomes in lung cancer when patients are treated with checkpoint inhibitors^87^. These data suggest that subclonal neo-antigens might impede cytotoxic immune responses against neo-antigens that are present in every tumour cell.

Other hazards faced by neoplastic cells include the accumulation of waste products in their micro-environments^67,69,88,89^. This may include lactic acid and lactate build-up from glycolysis^88,90^ as well as reactive oxygen species from excessive cellular proliferation^91^. Methylglyoxal^92,93^, nitric oxide^94,95^ and advanced glycation end products^96,97^ have also been implicated as toxic waste products in cancer microenvironments.

The role of the microbiome in cancer is complicated and largely unknown. While some microorganisms may promote tumours^98,99^, others have antitumour effects^98^, enhancing the efficacy of chemotherapy^100^. Thus, micro-organisms may act as both resources and hazards for neoplastic cells.

### Measuring hazards

The current best measures of hazards for a neoplastic cell depend on measures of immune predation (TABLE 1). There is a large literature on the association between infiltrating lymphocytes and favourable prognosis in cancer^73,75–83^. In addition, a pan-cancer analysis revealed T cell signatures to be broadly favourable prognostic markers across 25 cancer types^101^. Galon and colleagues have found that a signature of activated T cells from bulk tumour samples is also strongly predictive of favourable survival^76–78,83^. Yuan and colleagues have shown that haematoxylin and eosin images can be computationally analysed to identify neoplastic cells, fibroblasts and lymphocytes and, furthermore, that patients with breast cancer who show colocalization of neoplastic cells with lymphocytes in the tumour have a better prognosis than patients with tumours in which the lymphocytes are separated from the neoplastic cells^75^. This is based on a standard ecological statistic, the Morisita–Horn index^102^, for quantifying statistically significant colocalization in order to detect ecological interactions (in this case, predation). These results suggest that immune predation is a major form of hazard for a neoplastic cell, and measures of that predation should be a central component of the ecological index.

While much research has investigated the potentially toxic effects of low pH (REFS 103,104), fewer studies have examined the fitness consequences to cancer cells from various metabolites. Future research should determine the effects of different concentrations of putative toxic metabolites on cancer cell survival and proliferation in both cell culture experiments and mouse models. Measurements of anticancer drug concentrations in the tumour are also likely to quantify important hazards for the neoplastic cells. In addition, the microbiome (including the virome) of tumours can be surveyed to reveal microbial hazards for the neoplastic cells^105^.

### Resources

Resources, including oxygen, glucose, micronutrients, survival signals, growth signals and space, are also critical to the future behaviour of a tumour. Surprisingly little is known about the interactions between cell metabolism and the availability of key resources, which ecologists term the organism’s ‘foraging ecology’. Almost all cancers rely on glycolytic as opposed to aerobic metabolism, suggesting that resources can select for tumour phenotypes^106,107^. From nature, we know that selection favours feeding behaviours that balance speed, efficiency and safety^108^. There must be strong selection for cancer cells to do the same (for example, through upregulation of transporters such as glucose transporter type 1, erythrocyte/brain (GLUT1, also known as SLC2A1)^109^). Measuring which resources limit the population size and proliferation of neoplastic cells would allow researchers to identify some of the strongest selective pressures on the tumour and to predict how it will change in the future. This approach would also provide targets for further reducing the evolvability of the neoplasm by lowering the carrying capacity of its microenvironment.

In the broader ecological literature, consumer–resource theory^110^ shows that resource supply, depletion and availability affect population growth rates, population sizes and competition between different species (that is, distinct clonal lineages). Resource supply represents the rate at which new resources enter the system (in this case, the tumour) and the rate at which resources become available through nutrient cycling within the system. The aggregate consumption of all cells depletes the resources, typically to levels much lower than experienced by normal tissues^111^. In fact, glucose becomes depleted below levels detectable by most analyses^112^. However, in some cases, immune predation and fluctuations in resource supply can prevent the complete exploitation of resources^113,114^, leaving patches of residual resources available for future exploitation^115^.

The potential resources for a tumour include the contents of plasma and the metabolites synthesized and secreted by the normal cells of the tumour and its microenvironment. Hence, the list includes proteins (albumins, globulins and fibrinogens), glucose, amino acids, fatty acids, hormones, electrolytes, oxygen and trace elements. The functional response and the value of the resources to the consumer are dictated by nutritional relationships^116^. In some cases, lack of a resource may trigger stasis, but in others, it may lead to cell death or dispersal^117^. At the moment, there are many open questions about the intratumoural cycle of critical nutrients other than carbon and nitrogen (that is, phosphate, iron, copper, etc.)^118^. These nutrient cycles may contain valuable therapeutic targets.

Some resources, particularly growth and survival signals, may be provided by the neighbouring stromal cells^119,120^. Nutrients may also be provided by the stroma. Pyruvate and lactate can be supplied to cancer cells by activated fibroblasts^121,122^, and fatty acids may be supplied by activated adipocytes^123,124^. Tumour and stroma only come into physical contact when the basement membrane is breached by malignant neoplastic cells. At this stage, cancer cells can directly interact with cancer-associated fibroblasts, which are known to play a key role in the regulation and development of tumours, especially solid tumours^120,125^. In this secretory reactive state, fibroblasts facilitate not only cancer growth and progression^126,127^ but also treatment resistance^128^. In addition, their presence in a tumour has been correlated with poor outcomes^129^.

Other resources must be delivered through the vasculature. Folkman made the crucial link between angiogenesis and tumour invasion and metastasis, realizing that preventing new vessels from forming could be a simple way to inhibit further tumour growth^130,131^. The presence in many tumours of necrosis and hypoxia, which are major drivers of angiogenesis, attests to the importance of resource limitation in tumours. Furthermore, there is evidence that necrosis is a prognostic factor in many cancers^132^.

The effects of resources on the evolution of a tumour are not defined simply by their supply, depletion and availability. Resource diversity may also be important. Whether resources are uniform across space or heterogeneous (‘patchy’ or exhibiting gradients) makes a difference^67,133^. Patchy resources (and hazards) create multiple habitats (for example, rich and sparse regions) that may select for different clones that can survive in those regions and may be differentially responsive to (and differentially exposed to) therapies. Furthermore, we and others have shown that if those patchy resources change over time, then there is selective pressure on cells to move to escape regions of scarce resources and exploit transient regions of more plentiful resources^67,113,114,134–136^. Thus, ecological theory predicts that heterogeneous resources should select for invasion and metastasis^134,135^, and there is evidence to support that prediction in cancer^137–143^. Verduzco and colleagues found that intermittent exposure of some cell lines to hypoxia selected for increased resistance to a variety of chemotherapies, including etoposide, docetaxel and methotrexate, compared with unselected controls^144^. In addition, resource gradients often lead to rapid evolution, as organisms that are able to invade more stressful environments can escape competition and flourish^145^. Much needs to be learned about resource heterogeneity, consumer–resource dynamics and the foraging ecology of neoplastic cells.

### Measuring resources

Measuring resources (and hazards) requires the consideration of relevant spatial and temporal scales. It is not yet clear how to combine measures of the level of resources, their spatial variance and their stability over time into a single statistic.

There are various resources and methods to measure them that may be prognostically relevant (TABLE 1). The proportion of a tumour that is necrotic or poorly perfused may be read from standard positron emission tomography and computed tomography (PET–CT) images^146^ and through other measures of blood vessel density^147,148^. The degree and patchiness of hypoxia can also be assayed in FFPE samples with antibodies against carbonic anhydrase 9 (CA9) or hypoxia-inducible factor 1α (HIF1α)^115^ or via intravenous introduction of 2-(2-nitro-1-H-imidazol-1-yl)-*N*-(2,2,3,3,3- pentafluoropropyl) acetamide (EF5) and the subsequent measurement of its binding in the tumour tissue^149^. EF5 binding and related techniques have proved useful in the clinic for detecting regions of hypoxia, determining prognosis and measuring response to therapy^150^. While it is difficult to measure glucose concentration directly, an indirect measure may be made via immunohistochemistry staining for expression of GLUT1^115^. Measures of ATP may also be a good indirect measure of the amount of resources available to neoplastic cells^151^. Glutamine, pyruvate, lactate, fatty acids, calcium, potassium, phosphorus and various trace metals may also be limiting and important to measure, but this appears to be unexplored. Most of these measures will be limited to biopsy samples analysed *ex vivo* and thus will suffer the problems of spatial heterogeneity and sampling error.

In some cases, the problem of spatial heterogeneity and sampling error can be avoided through gross measures of resources from radiological images^152–154^ Radiographic images such as those obtained using PET–CT and magnetic resonance imaging (MRI) can provide valuable habitat data. In natural systems, there is usually a tight correlation between habitat and the types and characteristics of species inhabiting the habitat. Similarly, simply knowing the different habitat types within a tumour may be prognostic of the community of cancer cells and therapeutic outcomes. For instance, in glioblastoma, measures of fluid-attenuated inversion recovery (FLAIR), T1 and T2 from MRI examinations after gadolinium administration identified distinct habitats that correlated with therapeutic outcome, independent of tumour size^153^. Texture analysis of MRI scans has been used to identify spatial heterogeneity and regional variations that are associated with microenvironmental conditions, including cell density, tissue stiffness, blood flow and nutrient dispersion^152,154^. These may also be used to measure functional diversity (D) in tumours. Geographic information systems (GIS)^155–157^ and ecology^158^ provide a rich literature and a source of tools for analysing spatial resource information, but these are rarely utilized in cancer research^73,74^.

Standard histopathology can provide measures of T cell infiltration and vascular and lymphatic density^77^. Using digital pathology, Lloyd *et al.* investigated the spatial distributions of oestrogen receptor (ER) expression in relation to vascular density and tissue necrosis in breast cancer histology specimens, revealing considerable regional variations in cancer proliferation phenotypes accompanied by vascularity and immune response^115,159^. Yuan and colleagues also used digital pathology to analyse the spatial relationships between fibroblasts and neoplastic cells^160^. We have summarized the statistics and assays for measuring diversity, change over time, hazards and resources in TABLE 1.

## Categories of tumours

The future behaviour of a tumour depends on both its evolutionary potential (the Evo-index) and the selective pressures on the tumour (the Eco-index). A highly evolvable tumour may or may not evolve immune evasion depending on whether the immune system is imposing a strong selective pressure on the tumour. By contrast, an immune response may or may not lead to immune evasion depending on the evolvability of the tumour. Thus, both the evolution and ecology of a tumour must be considered in predicting cancer outcomes. We therefore propose to combine the Evo- and Eco-indices to classify tumours. Dichotomizing each evolutionary and ecological factor of the Evo- and Eco-indices into high and low values would produce 16 possible types of tumour (TABLE 2).

In order to classify a tumour, investigators will first need to define and validate clinically relevant thresholds for dichotomizing diversity, change over time, hazards and resources (TABLE 1). For example, in Barrett oesophagus, Maley and colleagues found that the upper quartile of diversity statistics distinguished patients who are likely to progress to oesophageal adenocarcinoma^12–14^. Once those thresholds are validated, a tumour would be measured for each of the four evolutionary and ecological factors to determine which of the 16 types it falls into. For example, if a tumour was below the thresholds for all four factors (that is, a D1Δ1H1R1 tumour), it would be a type 1 tumour.

### A roadmap for improvements

We are not yet in a position to specify which measures and thresholds should be used to determine the D#Δ# or H#R# type of a tumour. Initial studies should test if these classifications significantly predict clinical outcomes and which evolutionary and ecological measures provide independent predictive value. They should also test if there are measures that can apply across cancer types or if they have to be uniquely defined for specific organs or tumour subtypes. Future studies should test alternative measures of diversity, change over time (BOX 2), hazards and resources to help standardize useful metrics for the classifications. They should also quantify the improvements to prognosis gained by sampling multiple regions at multiple timepoints.

Box 2 **The future of the Evo-index**

Our framework for quantifying the evolvability of a neoplasm is based on the diversity within the tumour and how that diversity changes over time. Diversity and genetic change over time are the easily observable results of the underlying evolutionary dynamics. A future evolutionary index (Evo-index) may be based on the parameters that determine the rates of evolution^15^:

- Mutation rate^17,178^
- Population size of the self-renewing neoplastic cells (also known as ‘cancer stem cells’), which are the units of evolutionary selection in cancer^3^
- Generation time of the self-renewing neoplastic cells
- Selective coefficients^17,179^ or clonal expansion rates^14^
- Heritability of selectively advantageous phenotypes

Most of these parameters are currently difficult to measure. However, there is already good evidence that the number of self-renewing cells in a tumour is associated with adverse outcomes^180,181^, that self-renewing cell frequency increases with progression^182,183^ and that self-renewal signalling pathways are actionable and effective targets for therapy^184,185^. This is probably true for all types of tumour. Assaying self-renewing cells functionally (by xenotransplantation) is difficult, but quantifying stem cell signatures is possible. However, stem cell phenotypes are not stable and can be modulated both by genetic changes and (epigenetically) by ecological conditions (for example, hypoxia)^3^, suggesting that the importance of any one parameter is also a function of its heritability.

The ecology of a tumour affects its evolution, and the evolution of the cells in a tumour change their ecology. Neoplastic cells evolve genomic instability^161^, generating neo-antigens as well as adaptations, such as recruitment of resources, through activating fibroblasts^162^ and neo-angiogenesis^161^. Evolution of neo-antigens triggers immune predation, which may reduce diversity and select for immune evasion^163^. High levels of extrinsic mortality and resources select for rapid proliferation with little investment in somatic maintenance^71^. These interactions imply that not all possible combinations of ecological and evolutionary measurements are equally likely. We will probably be able to drop some of the 16 possible tumour types in TABLE 2 and focus on the subset of classes that present in the clinic.

**Figure 4:**
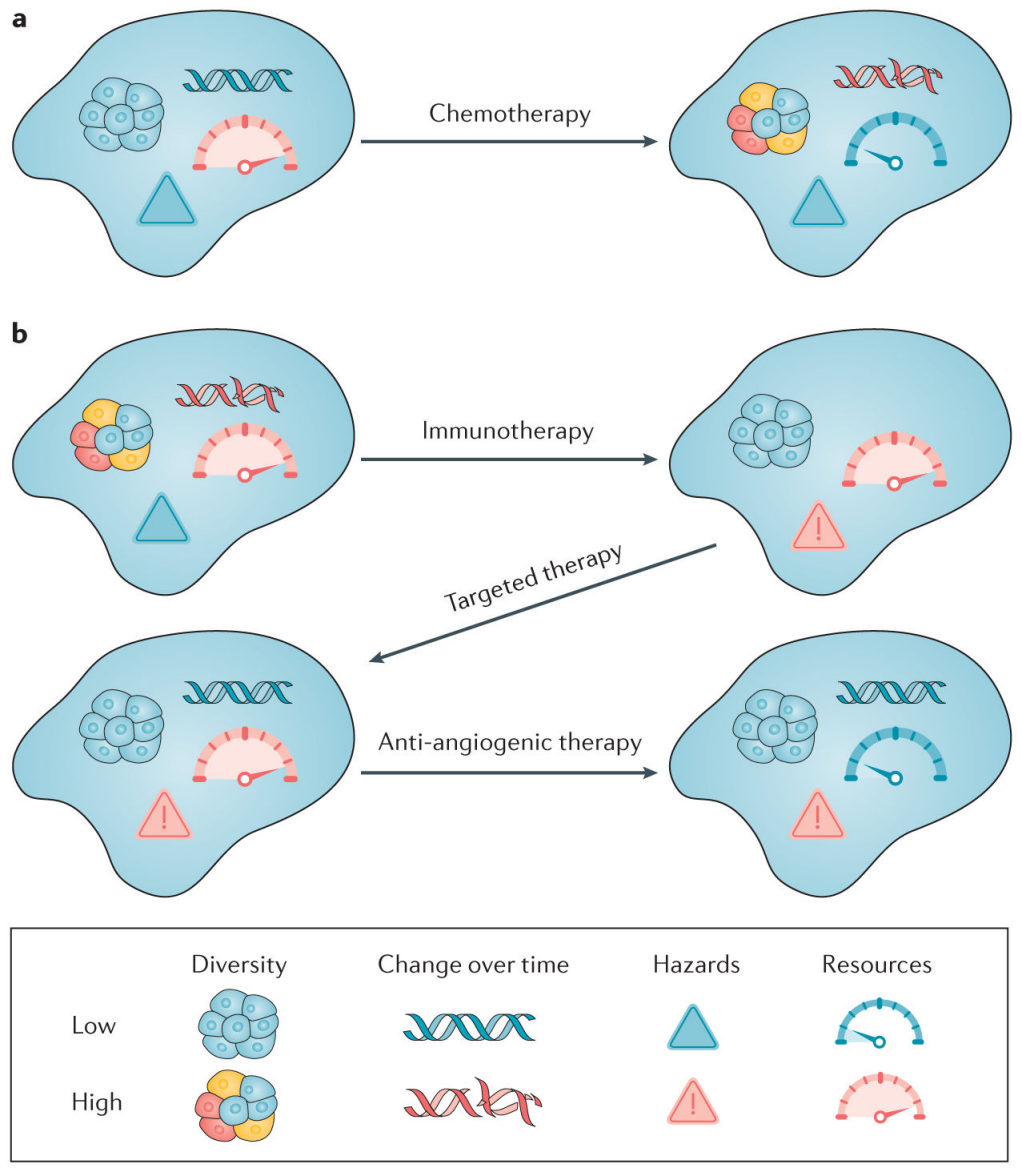
Changing the evolutionary class of a tumour through interventions With the classification system outlined in TABLE 2, we could examine how different interventions move tumours between categories. **a** | In this example, chemotherapy can be mutagenic and can select for hypermutator clones, generating new clones and more diversity^40,186,187^. It can also kill endothelial cells and thus have an anti-angiogenic effect^188^, resulting in a tumour (type 13) with one of the worst predicted prognoses. This may partly explain why tumours that recur after chemotherapy are so difficult to control. **b** | Immunotherapy, if successful, may increase the predation hazards to the tumour and perhaps select for a subclone, reducing diversity. Targeted therapy, unlike chemotherapy, probably does not cause significant DNA damage and may further genetically homogenize the tumour. Anti-angiogenic therapy is designed to restrict the resources of the tumour. At the end of this example sequence, the tumour is in the most manageable, least evolvable category (type 3 in TABLE 2). Of course, chemotherapy, immunotherapy and targeted therapy may have different effects depending on the details of those therapies and their interaction with the clones in the tumour and their ecosystem.

The framework for a classification system that we have proposed could be incorporated into clinical trials, which could allow us to gather data on how the different types of evolving tumour respond to different types of intervention (FIG. 4). Clinical trials could then be developed to stratify treatment of patients based on the Evo- and Eco-indices of their tumours. We could use the results to develop guidelines for best practice in managing cancers.

## Vision of the future

In the future, the pathology report for a neoplasm could include its Evo-index and Eco-index classifications. Ideally, these classifications would provide ‘chessboard’-like scenarios where, based on the current evolutionary class of a tumour, one could anticipate how the tumour type will change with different possible therapeutic moves (FIG. 4). Clinicians would then be able to choose appropriate interventions for the evolvability of those neoplasms and would also be able to track whether the neoplasms change substantially in response to interventions. A D1Δ1 tumour or even a D1Δ2 tumour would be a prime candidate for aggressive therapy with curative intent. In fact, a D1Δ1 tumour may be so evolutionarily indolent as to not require any form of intervention. On the other hand, a D2Δ2 tumour is likely to have multiple resistant sub-clones present at diagnosis, and future clinical trials should test if such a tumour can be managed through strategies that minimize the expansion of resistant sub-clones by exploiting their disadvantage in competition with sensitive subclones^164^. A legitimate clinical strategy might be to down-stage a tumour from a highly evolvable one to a much more clinically manageable class that could be contained in a non-lethal state indefinitely (FIG. 4b). If validated, the Evo- and Eco-indices could be used as surrogate measures for overall survival or disease-free survival.

## Conclusions

The evolutionary biology of cancer is, clinically, in a similar state to psychiatry in the nineteenth century. At that time, there was no standard classification system for mental illness used by practitioners. Without such a classification system, it was difficult to even talk about the illness, let alone make progress, as a common language was lacking. With the American Medical Association’s Standard Classified Nomenclature of Disease published in 1933 (REF. 165) and the first Diagnostic and Statistical Manual of Mental Disorders published in 1952 (REF. 166), no matter how flawed they were, diagnoses of mental disorders became standardized, which facilitated studies to refine both the classifications as well as the treatment of those disorders. Studies based on the same classification system were then comparable, which further facilitated meta-analyses and overall progress in the field.

We have diagnostic categories for types of tumour based on their tissue of origin and staging, as well as some molecular markers, but we have lacked a system for classifying the evolvability and ecology of a tumour, which help determine how it will respond to interventions and how it might best be managed. Evolutionary oncology requires a shared lexicon upon which to base discovery. We reached consensus on the proposed framework for a classification system to characterize evolutionary differences between tumours that is applicable across all cancer types. Importantly, an evolutionary classification system will facilitate future efforts to study this fundamental property of tumours to reveal implications for treatment.

## Figures and Tables

**Table 1:** Measures and assays for the factors that go into the Evo- and Eco-indices

Icon	Factor	Statistics	Assays
High 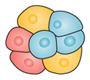 Low 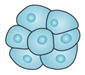	Diversity (D)	Divergence^12–14,46^ Number of clones (richness)^6,12–14^ Shannon index^12–14^ Simpson’s index^12,13^ Functional diversity^115,169,170,61–63^ Phylogenetic trees^20^	Whole-exome and whole-genome sequencing Multi-region sequencing SNP arrays Methylation arrays FISH Single-cell DNA and RNA sequencing Cell-free DNA sequencing^19^ RNA-Seq Proteomics Radiology
High 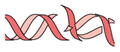 Low 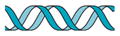	Change over time (Δ)	Mutation rates^17,178^ Estimates of selection^17,179^ Clonal expansion rates^14^ *F*_ST_ (REF. 60) Nei’s standard genetic distance^57,58^ Change in above diversity statistics	Longitudinal sampling Whole-exome and whole-genome sequencing Cell-free DNA analysis^19^
High 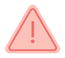 Low 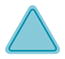	Hazards (H)	Abundance of infiltrating lymphocytes^82,83^ Morisita–Horn index of colocalization of cancer cells and lymphocytes^75^ Signatures of immune activation^82,83,101^ Density of pathogenic microorganisms^99^ Prevalence of microbial virulence genes^105^	Automated image analysis Immunohistochemistry RNA-Seq 16S rRNA sequencing
High 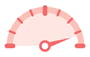 Low 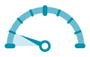	Resources (R)	Degree of hypoxia^146,149^ Density of blood vessels^147,148^ Colocalization of cancer cells with fibroblasts^160^ Concentration of ATP^151^, glucose and other nutrients Blood flow^152,154^	Automated image analysis Immunohistochemistry MRI or PET–CT scans Intravenous induction of EF5 Luciferase luminescence Mass spectrometry

Eco-index, ecological index; EF5, 2-(2-nitro-1-H-imidazol-1-yl)-N-(2,2,3,3,3-pentafluoropropyl) acetamide; Evo-index, evolutionary index; FISH, fluorescence *in situ* hybridization; *F*_ST_, fixation index; MRI, magnetic resonance imaging; RNA-Seq, RNA sequencing; rRNA, ribosomal RNA; PET CT, positron emission tomography and computed tomography; SNP, singe nucleotide polymorphism.

**Table 2:** An initial classification scheme

Type	Icon	Evo-index	Eco-index	Description
1	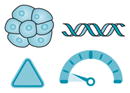	D1Δ1	H1R1	Like a desert, these tumours have few resources and little diversity. With low turnover, they are evolutionarily inert.
2	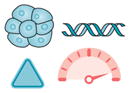	D1Δ1	H1R2	Much like normal tissue, these tumours have sufficient resources but evolve very slowly.
3	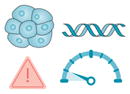	D1Δ1	H2R1	These tumours may have the best prognosis, with an immune response that probably helps to control the tumour, restricted resources and little capacity to evolve.
4	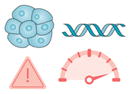	D1Δ1	H2R2	These tumours have ample resources but have also stimulated an antitumour immune response. However, they are otherwise evolutionarily inert.
5	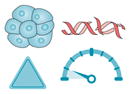	D1Δ2	H1R1	These tumours are genetically homogeneous but are changing over time, perhaps through population bottlenecks or selective sweeps that re-homogenize the tumour.
6	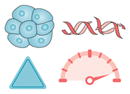	D1Δ2	H1R2	These tumours are changing over time, potentially through homogenizing selective sweeps of new clones. While they may grow rapidly, with ample resources, their genetic homogeneity may make them vulnerable to therapy.
7	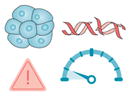	D1Δ2	H2R1	Predation by the immune system in these tumours may reduce genetic heterogeneity through selection against neo-antigens.
8	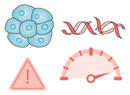	D1Δ2	H2R2	Natural selection may be driving the changes in these tumours and homogenizing them.
9	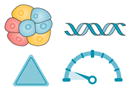	D2Δ1	H1R1	These tumours may be the result of the slow accumulation of clones over a long period of time or from exposure to mutagens.
10	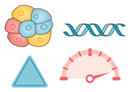	D2Δ1	H1R2	Like a garden, these tumours support a variety of clones, are well fed and are protected from hazards such as predation, but they change little over time.
11	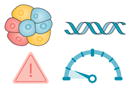	D2Δ1	H2R1	Accumulation of many mutations may have led to an immune response in these tumours, but they appear to be otherwise restricted in their growth and evolution.
12	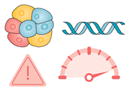	D2Δ1	H2R2	These genetically diverse tumours are changing only slowly, perhaps due to a low mutation rate or relatively weak selective pressures.
13	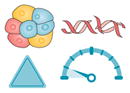	D2Δ2	H1R1	These tumours are evolving rapidly, generating and maintaining new clones at a high rate. They are probably under selective pressure for the ability to survive and proliferate with scarce resources or otherwise escape these resource constraints.
14	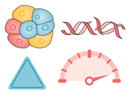	D2Δ2	H1R2	With potentially the worst prognosis, these genetically diverse tumours are evolving rapidly and have plenty of resources. They should have the highest capacity to evolve in response to interventions or other changes in their environment.
15	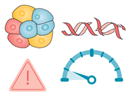	D2Δ2	H2R1	These rapidly evolving and diverse tumours are under the dual selective pressures of resource limitations and immune predation.
16	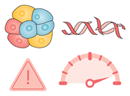	D2Δ2	H2R2	Like a rainforest, these genetically diverse tumours are changing rapidly, with a constant churn of new clones evolving and others going extinct. Resources are abundant, although they are probably being consumed rapidly, and predation from the immune system is extensive.

D, diversity; Δ, genetic, epigenetic or phenotypic change over time; Eco-index, ecological index; Evo-index, evolutionary index; H, hazards; R, resources.

## Abbreviations

Clones: Sets of cells that share an alteration of interest due to descent from a common ancestor cell.
Selective sweep: The spread of a mutation through a population due to natural selection.
Phenotypic diversity: The variety of different cellular states present in a population of cells.
Functional diversity: The variety of life history strategies present in a population of cells.
Genetic drift: Change in allele frequencies due to sampling error of gene copies from one generation to the next. Genetic drift is stronger in smaller populations.
Muller’s ratchet: Accumulation of deleterious mutations in asexual populations. This accumulation is irreversible because in asexual populations, deleterious mutations cannot be purged through recombination.
Nei’s standard genetic distance (*D*_S_).: A measure of the genetic divergence between species or populations given their respective allele frequencies. When the mutation rate is constant, *D*_S_ increases linearly with time, from zero to infinity. In a multiregional or longitudinal sequencing study, it would quantify the amount of genetic divergence between two regional or temporal biopsy samples.
Jaccard similarity coefficient: The proportion of species or clones that are present in both regions compared with the number of species or clones observed in the union of both regions.
UniFrac: A measure of the difference between biological communities that takes into account the phylogenetic distances (relatedness) between community members as well as their relative abundances.
Fixation index (*F*_ST_).: A population genetic measure that estimates the proportion of global genetic variability that can be explained by population structure. In a multiregional sequencing study, it would quantify how much of the intratumoural heterogeneity is due to differences between regional biopsy samples.
Life history strategies: Relative investments in and mechanisms of growth, reproduction and survival of specific organisms or cells.
Morisita–Horn index: A statistic for measuring the extent to which two species tend to co-occur in the same locales.
Selective coefficients: The relative differences in fitness between genotypes.
